# Extremes of both weight gain and weight loss are associated with increased incidence of heart failure and cardiovascular death: evidence from the CANVAS Program and CREDENCE

**DOI:** 10.1186/s12933-023-01832-5

**Published:** 2023-04-29

**Authors:** Giulia Ferrannini, Carol Pollock, Andrea Natali, Yshai Yavin, Kenneth W. Mahaffey, Ele Ferrannini

**Affiliations:** 1 Department of Medicine Solna, Karolinska Institutet, Eugeniavägen 27, Solna, S1:02, 171 64 Stockholm Sweden; 2 Kolling Institute of Medical Research, Sydney Medical School, University of Sydney, Camperdown, Australia; 3 Department of Clinical and Experimental Medicine, University of Pisa, Pisa, Italy; 4 Janssen Research & Development, LLC, Welsh & McKean Rds, Spring House, PA USA; 5 Stanford Center for Clinical Research, Department of Medicine, Stanford University School of Medicine, Stanford, CA USA; 6 CNR Institute of Clinical Physiology, Via Savi 12, Pisa, 56126 Italy

**Keywords:** Weight change, Obesity, Heart failure hospitalization, Cardiovascular death, Canagliflozin, Type 2 diabetes

## Abstract

**Background:**

Obesity is an independent risk factor for cardiovascular disease (CVD) in patients with type 2 diabetes (T2D). However, it is not known to what extent weight fluctuations might be associated with adverse outcomes. We aimed at assessing the associations between extreme weight changes and cardiovascular outcomes in two large randomised controlled trials of canagliflozin in patients with T2D and high cardiovascular (CV) risk.

**Methods:**

In the study populations of the CANVAS Program and CREDENCE trials, weight change was evaluated between randomization and week 52–78, defining subjects in the top 10% of the entire distribution of weight changes as *gainers*, subjects in the bottom 10% as *losers* and the remainder as *stable*. Univariate and multivariate Cox proportional hazards models were used to test the associations between weight changes categories, randomised treatment and covariates with heart failure hospitalisation (hHF) and the composite of hHF and CV death.

**Results:**

Median weight gain was 4.5 kg in *gainers* and median weight loss was 8.5 kg in *losers*. The clinical phenotype of *gainers* as well as that of *losers* were similar to that of *stable* subjects. Weight change within each category was only slightly larger with canagliflozin than placebo. In both trials, *gainers* and *losers* had a higher risk of hHF and of hHF/CV death compared with *stable* at univariate analysis. In CANVAS, this association was still significant by multivariate analysis for hHF/CV death in both *gainers* and *losers* vs. *stable* (hazard ratio – HR 1.61 [95% confidence interval - CI: 1.20–2.16] and 1.53 [95% CI 1.14–2.03] respectively). Results were similar in CREDENCE for *gainers* vs. *stable* (adjusted HR for hHF/CV death 1.62 [95% CI 1.19–2.16])

**Conclusions:**

Extremes of weight gain or loss were independently associated with a higher risk of the composite of hHF and CV death. In patients with T2D and high CV risk, large changes in body weight should be carefully assessed in view of individualised management.

**Trials registration:**

CANVAS ClinicalTrials.gov number: NCT01032629. CREDENCE ClinicalTrials.gov number: NCT02065791

**Supplementary Information:**

The online version contains supplementary material available at 10.1186/s12933-023-01832-5.

## Background

Epidemiological evidence uniformly supports the notion that obesity is an independent risk factor for cardiovascular disease (CVD) [1]. A meta-analysis of randomised controlled trials (RCT) of dietary interventions targeting weight loss in adults with obesity showed that weight-reducing diets may decrease premature all-cause mortality [2]. However, several studies in patients with CVD reported conflicting results: in two large cohorts a reduction of more than four body mass index (BMI) units (~ 10 kg) from before to after a myocardial infarction (MI) was associated with increased mortality compared with stable weight [3]. In the ProActive trial, investigating the effect of pioglitazone in patients with type 2 diabetes (T2D) and CVD, overweight and obese patients had a lower mortality compared to patients with normal weight, and weight loss but not weight gain was associated with increased mortality and morbidity [4]. This might be due to reverse causation, indicating a better nutritional status and cardiometabolic fitness in patients with higher BMI and CVD [5]. On the other hand, a single BMI assessment, although commonly used, might not be an adequate indicator of body composition [6]. Weight changes might be more compelling in studying the association between BMI and CVD outcomes [7, 8], but their impact on CVD outcomes is less clear and has not been analysed separately, nor is it known how their effect may be modulated by the presence of T2D.

Aim of this work was to test whether large, time- or treatment-related weight changes may impact major CVD outcomes independently of body size itself. To this end, we explored data from the CANVAS Program, a RCT investigating the effect of canagliflozin, a sodium-glucose cotransporter-2 inhibitor (SGLT2i), on cardiovascular (CV) outcomes in patients with T2D. We sought replication in the data of CREDENCE, an RCT of canagliflozin in patients with T2D and renal impairment, in whom the main endpoint was progression of diabetic kidney disease (DKD). Although treatment with SGLT2i is typically associated with weight loss [9], this change is highly variable in size and is not clearly dependent on drug-induced glycosuria [10].

## Methods

### Study populations

*CANVAS Program* The CANVAS Program, which integrated the CANVAS and CANVAS-R trials, investigated the effects of canagliflozin on CV, renal and safety outcomes in 10,142 patients with T2D and either established CV disease or at high CV risk, with a mean follow-up time of 188 weeks. Details of the CANVAS Program design have been published [11, 12]. In brief, participants in CANVAS were randomised (1:1:1) to receive canagliflozin 300 mg, canagliflozin 100 mg, or placebo, and participants in CANVAS-R were randomised (1:1) to receive canagliflozin 100 mg, with optional uptitration to 300 mg starting from week 13, or placebo. Adjudicated outcomes in the CANVAS Program were major adverse CV events (MACE – a composite of death from CV causes, nonfatal myocardial infarction - MI, or nonfatal stroke), death from any cause, death from CV causes, hospitalised heart failure hHF), the composite of death from CV causes and HF, and a renal composite outcome, comprising a > 40% reduction in estimated glomerular filtration rate (eGFR) sustained for at least two consecutive measures, the need for renal-replacement therapy (dialysis or transplantation), or death from renal causes (defined as death with a proximate renal cause), and progression to macroalbuminuria [13]. Further detail on the CANVAS Program is publicly available via the Yale University Open Data Access Project (http://yoda.yale.edu/).

*CREDENCE* The CREDENCE trial enrolled 4401 patients with type 2 diabetes, CKD (eGFR ≥ 30 to < 90 mL/min/1.73 m^2^) and albuminuria (urine albumin-to-creatinine ratio [UACR] > 300 to ≤ 5000 mg/g) who were randomised (1:1) to canagliflozin 100 mg or placebo, with stratification by baseline eGFR (30–44, 45–60, and 60–90 mL/min/1.73 m^2^) [14]. The primary composite outcome was end-stage kidney disease (dialysis for at least 30 days, kidney transplantation, or an eGFR of < 15 mL/min/1.73m^2^ sustained for at least 30 days), doubling of the serum creatinine level from baseline sustained for at least 30 days, or death from renal or CV disease. Secondary outcomes undergoing sequential hierarchical testing were, in order: (i) a composite of CV death or hHF; (ii) a composite of CV death, MI, or stroke; (iii) hHF alone; (iv) a composite of end-stage kidney disease, doubling of the serum creatinine level, or renal death; (v) CV death; (vi) death from any cause; (vii) a composite of CV death, MI, stroke, hHF or hospitalization for unstable angina.

The CREDENCE trial was stopped early after a planned interim analysis, with a final median follow-up of 2.6 years.

### Body weight measurements

For the present investigation, patients with available data on weight at weeks 52 and 78 were considered (a total of 8,656 individuals). The weight change from baseline at 52 weeks and the weight change from baseline at 78 weeks were averaged.

We hereinafter define subjects in the top 10% of the entire distribution of weight changes as *gainers*, subjects in the bottom 10% of the distribution as *losers* and the remainder of the cohort as *stable*.

### Statistical analysis

Data are presented as mean ± standard deviation (SD) for variables with a normal distribution or median [interquartile range (IQR)] for variables with a skewed distribution according to the Shapiro–Wilk test. Differences in baseline characteristics between *gainers* and *stable*, between *losers* and *stable* and between patients assigned to canagliflozin vs. placebo within each weight change category were assessed by two-way ANOVA for continuous variables and by the chi-square test for categorical variables. Differences between the subgroups randomized to canagliflozin vs. placebo were computed by Cochran-Mantel-Haenszel test. Univariate and multivariate Cox proportional hazards models were used to test the association of weight changes categories, randomised treatment and different covariates with the outcomes of interest, i.e., hHF, the composite of hHF and CV death, MACE and non-fatal MI in both trials. The associations were expressed as hazard ratios (HR) − 95% confidence intervals (CI) and calculated for 1 SD for age and baseline weight, which had a normal distribution, and 1 log unit for UACR, which had a skewed distribution; all other variables were binary. Interaction between weight loss category and treatment was tested for both hHF and hHF + CV death in both datasets. In multivariate models, adjustments were performed for those clinical parameters that were significantly different between *gainers* or *losers* and *stable* subjects (namely, sex, age, baseline weight, UACR, smoking, use of diuretics, statins, antithrombotics, insulin, metformin, sulphonylureas and GLP1 receptor agonists) in addition to canagliflozin treatment.

CV medications and canagliflozin treatment). Event curves for the time-to-first hHF in different weight categories were computed by the Kaplan–Meier estimator and compared by the log-rank test. All analyses were performed using JMP Pro 15.2.0®.

## Results

### CANVAS

**Fig. 1:**
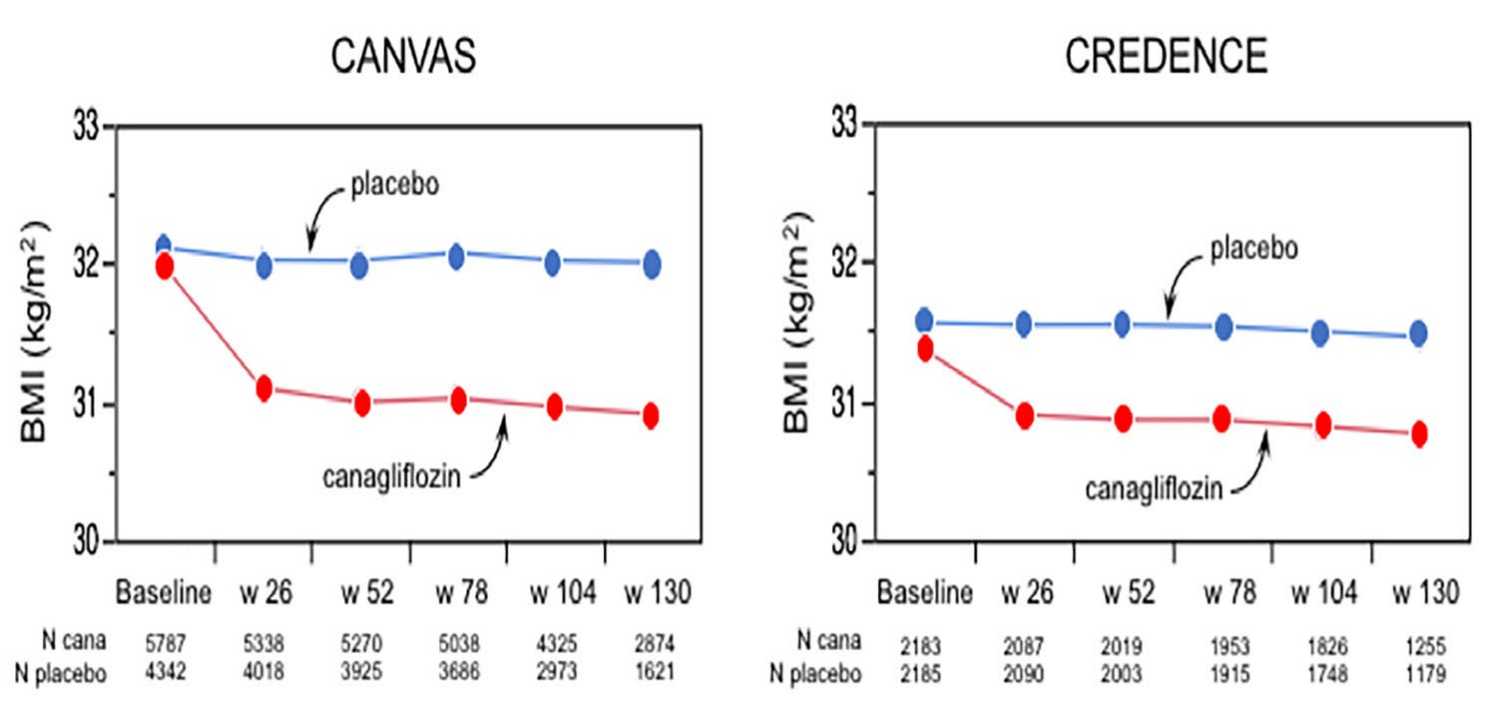
Body mass index (BMI) at baseline and follow up in CANVAS and CREDENCE. Dots are least-squares means. In both datasets, *p* < 0.0001 for the interaction of BMI and time by repeated-measures ANOVA

**Fig. 2:**
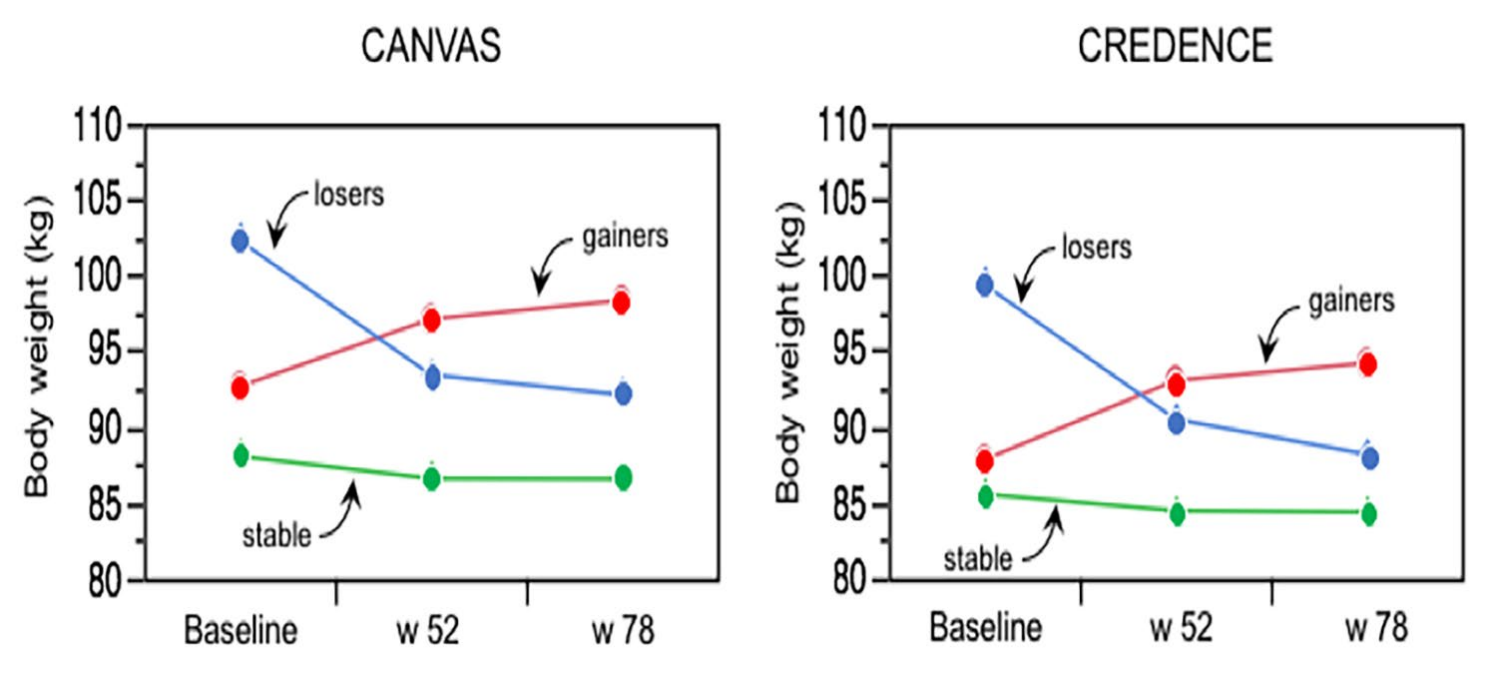
Body weight at baseline at week 52 and 78 in the three weight change categories in CANVAS and CREDENCE. Dots are least-squares means. In both datasets, *p* < 0.0001 for body weight differences between *gainers* vs. *stable* and *losers* vs. *stable* by repeated-measures ANOVA.

BMI at baseline in the whole cohort averaged 31.9 kg/m^2^ and was essentially stable in the placebo arm throughout follow-up; in the canagliflozin arm it was decreased at week 26 and stabilized thereafter (Fig. 1**)**; the corresponding changes in body weight are depicted in Fig. 1 from ref [11]. In the whole cohort, weight change between randomisation and week 52–78 averaged − 1.5 kg (− 2%), with a wide dispersion (ranging from − 39.4, − 36%, to + 23.1 kg, + 29%) and a significantly non-normal distribution (*p* < 0.01 by Shapiro–Wilk test). Lower boundary of weight gain in *gainers* was > 2.9 kg and that for weight loss in *losers* was > 6.5 kg. The clinical phenotype of these three groups is shown in Table 1 by treatment arm. As expected, percentage of participants in the canagliflozin arm was half that of placebo arm among *gainers*, 38% higher among weight stable, and more than three times higher among *losers*. Age was younger in *gainers* and older in *losers*, with small differences between canagliflozin and placebo. Median weight gain was 4.5 kg (+ 5%) in *gainers* and median weight loss was − 8.5 kg (− 9%) in *losers*; in weight *stable* and *losers*, weight loss was higher with canagliflozin than placebo (*p* < 0.0001). Notably, body weight was higher at baseline and at the end of the study in both *gainers* and *losers* as compared to weight *stable* (all *p* < 0.0001) (Fig. 2). Otherwise, diabetes duration, haemoglobin A_1c_ (HbA_1c_), high-density lipoprotein (HDL)-cholesterol, prior history of CVD, DKD, and heart failure were essentially balanced across groups, while smoking was more prevalent among *losers*. With regard to CV therapy, statins, renin-angiotensin-aldosterone-system (RAAS) inhibitors, and ß-blockers were used similarly in all groups; use of loop or non-loop diuretics and antithrombotics was higher in *losers vs. stable*. As for antidiabetic treatment, use of insulin was more prevalent – and use of metformin was less prevalent – among *gainers*; sulphonylureas were less common among both *gainers* and *losers* while very few patients were on a glucagon-like peptide-1 receptor agonist (GLP-1 RA).

**Table 1:** Clinical and metabolic characteristics of the CANVAS cohort by weight loss category and treatment*

Group (N, %)	Gainers (*G*) 867 (10)		Stable (*S*) 6936 (80)		Losers (*L*) 853 (10)		*p (G vs. S)*	*p (L vs. S)*	*p*_*Cana*_
**N (% of group)**	**Placebo** **580 (67)**	**Cana** **287 (33)**	**Placebo** **2894 (42)**	**Cana** **4042 (58)**	**Placebo** **184 (22)**	**Cana** **669 (78)**	**-**	**-**	**-**
**BMI change (kg/m** ^**2**^ **)**	**+ 1.6 [0.9]**	**+ 1.6 [0.9]**	**− 0.2 [1.1]**	**− 0.8 [1.2]**	**− 3.0 [1.2]**	**− 3.0 [1.3]**	**< 0.0001**	**< 0.0001**	**< 0.0001**
**Weight change (kg)**	**+ 4.5 [2.4]**	**+ 4.5 [2.5]**	**− 0.6 [3.0]**	**− 2.2 [3.4]**	**− 8.2 [3.1]**	**− 8.7 [3.4]**	**< 0.0001**	**< 0.0001**	**< 0.0001**
**Percent weight change**	**+ 5.0**	**+ 5.1**	**− 0.7**	**− 2.6**	**− 8.9**	**− 8.9**	**< 0.0001**	**< 0.0001**	**< 0.0001**
*Clinical phenotype*									
Sex (% M)	63	64	63	64	63	68	ns	ns	< 0.0001
Age (years)	62 ± 8	61 ± 8	64 ± 8	63 ± 8	64 ± 8	63 ± 9	< 0.0001	0.0007	0.0060
Baseline BMI (kg/m^2^)	32.7 ± 6.3	32.5 ± 6.5	31.5 ± 5.5	31.3 ± 5.5	35.9 ± 6.5	35.1 ± 6.1	0.0019	< 0.0001	ns
Baseline body weight (kg)	93 ± 22	92 ± 22	88 ± 19	88 ± 19	103 ± 21	102 ± 21	0.0009	< 0.0001	ns
eGFR (mL.min^-1^.1.73 m^-2^)	76 ± 22	77 ± 22	76 ± 20	77 ± 20	76 ± 21	79 ± 20	ns	ns	ns
Type 2 diabetes duration (years)	12 [9]	13 [9]	13 [10]	12 [9]	13 [11]	13 [10]	ns	ns	ns
HbA_1c_ (%)	8.28 ± 0.96	8.32 ± 1.03	8.22 ± 0.91	8.25 ± 0.94	8.25 ± 0.91	8.23 ± 0.92	ns	ns	ns
Systolic blood pressure (mmHg)	136 ± 16	135 ± 17	137 ± 15	136 ± 15	139 ± 17	137 ± 16	ns	ns	ns
UACR (mg/g)	14 [40]	16 [40]	12 [33]	12 [32]	14 [51]	12 [31]	0.0357	0.0150	ns
HDL cholesterol (mmol/L)	1.15 [0.42]	1.14 [0.38]	1.14 [0.37]	1.13 [0.38]	1.18 ± 0.33	1.18 ± 0.33	ns	ns	ns
Prior CVD (%)	67	67	66	64	69	66	ns	ns	ns
Prior MI (%)	31	29	29	28	36	30	ns	0.0489	ns
Prior HF (%)	15	19	15	14	23	13	ns	ns	ns
Smokers (%)	16	16	18	17	21	23	ns	0.0005	0.0008
Use of loop or non-loop diuretics (%)	47	47	45	42	48	48	0.0451	0.0184	0.0097
Use of antithrombotics (%)	76	72	74	72	82	75	ns	0.0162	0.0322
Use of statin (%)	73	70	76	75	76	76	0.0211	ns	ns
Use of RAAS inhibitors (%)	78	81	81	81	79	81	ns	ns	ns
Use of ß-blockers (%)	57	55	53	53	70	51	ns	ns	ns
Use of insulin (%)	60	57	49	49	44	53	< 0.0001	ns	< 0.0001
Use of metformin (%)	73	72	80	78	77	79	0.0003	ns	0.0012
Use of sulphonylureas (%)	37	39	44	45	41	39	0.0002	0.0017	< 0.0001
Use of GLP-1 RA (%)	8	4	4	3	7	7	< 0.0001	< 0.0001	< 0.0001

*entries are mean ± standard deviation or median [interquartile range]. *p (G vs. S)* = Gainers vs. Stable and *p (L vs. S)* = Losers vs. Stable are computed by 2-way ANOVA or c^2^; *p*_*Cana*_ = Cana vs. Placebo is computed by Cochran-Mantel-Haenszel test

BMI, body mass index; eGFR, estimated glomerular filtration rate; UACR, urinary albumin-to-creatinine ratio; HDL, high-density lipoprotein; RAAS, renin-angiotensin-aldosterone-system; GLP-1 RA = glucagon-like peptide-1 receptor agonist; CVD, cardiovascular disease; HF, heart failure; MI, myocardial infarction

**Fig. 3:**
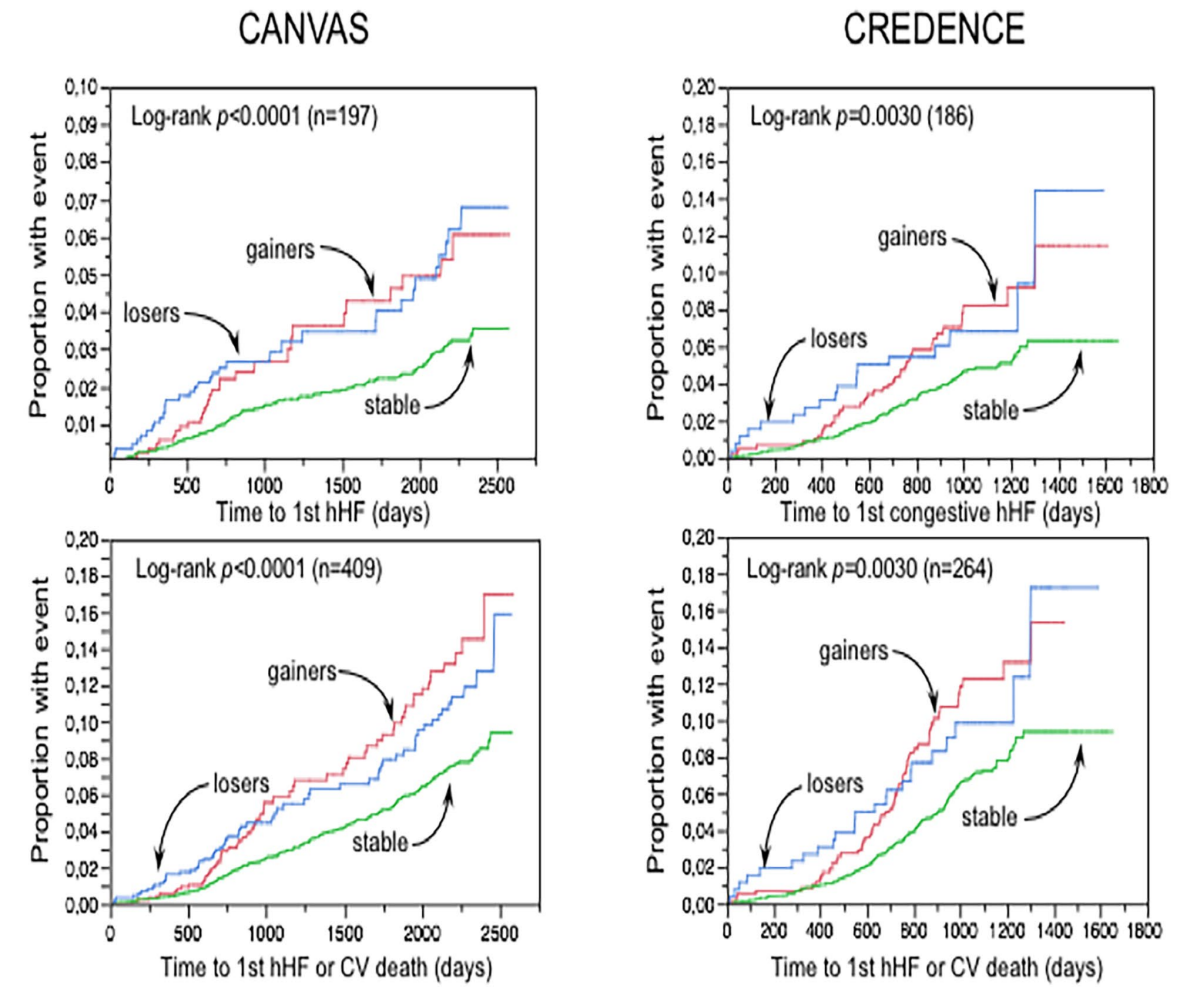
Kaplan–Meier plots of time to first hospitalised heart failure (hHF) and time to first hospitalised heart failure (hHF) and cardiovascular (CV) death in the three weight change categories in CANVAS and CREDENCE

The Kaplan-Meier functions for hHF of the three groups are depicted in Fig. 3; the proportion of patients with events was higher among both *gainers* and *losers* as compared to the *stable* group. In univariate analysis, the hazard ratio for the composite of hHF and CV death also was above unity for both *gainers* and *losers* as compared to weight *stable* (Fig. 3). In bivariate Cox models including group and treatment, the interaction of these two terms was *p* = 0.05 for hHF and *p* = 0.22 for hHF + CV death. In contrast, no associations of weight change category were found for events of MACE or non-fatal MI (Table 2).

**Table 2:** Univariate and multivariate association of weight changes with major CV outcomes in CANVAS. Entries are Hazard Ratio [95% confidence interval]

	hHF (n = 197)	hHF or CV Death (n = 409)	MACE (n = 712)	Non-fatal MI (n = 313)
*Univariate*				
**Gainers** ***vs*** **Stable**	1.85 [1.22–2.72]	1.84 [1.38–2.40]	1.26 [0.99–1.59]	1.25 [0.86–1.76]
**Losers** ***vs*** **Stable**	2.06 [1.40–2.95]	1.67 [1.26–2.18]	1.23 [0.97–1.54]	1.33 [0.93–1.83]
**Cana** ***vs*** **placebo**	0.60 [0.45–0.79]	0.76 [0.63–0.93]	0.91 [0.78–1.06]	0.86 [0.69–1.08]
*Multivariate*				
**Gainers** ***vs*** **Stable**	1.43 [0.93–2.13]	1.61 [1.20–2.13]	1.17 [0.91–1.47]	1.14 [0.78–1.62]
**Losers** ***vs*** **Stable**	1.67 [1.11–2.46]	1.53 [1.14–2.03]	1.18 [0.92–1.49]	1.27 [0.88–1.78]
**Cana** ***vs*** **placebo**	0.60 [0.45–0.80]	0.78 [0.64–0.96]	0.92 [0.78–1.07]	0.85 [0.68–1.08]
Sex (male)	1.09 [0.79–1.55]	1.02 [0.82–1.29]	1.27 [1.07–1.52]	1.63 [1.24–2.17]
Age (SD)	1.72 [1.47–2.03]	1.54 [1.38–1.72]	1.26 [1.17–1.37]	1.13 [1.00–1.28]
Baseline weight (SD)	1.45 [1.25–1.68]	1.21 [1.09–1.35]	1.09 [1.01–1.19]	1.09 [0.97–1.23]
Ln[UACR (mg/g)]	1.37 [1.27–1.48]	1.37 [1.29–1.44]	1.16 [1.11–1.22]	1.01 [0.94–1.09]
Smoking	1.03 [0.66–1.56]	1.19 [0.89–1.55]	1.01 [0.82–1.24]	0.86 [0.62–1.18]
Use of diuretics	2.25 [1.63–3.13]	1.72 [1.40–2.13]	1.05 [0.90–1.23]	1.03 [0.82–1.30]
Use of statins	1.04 [0.73–1.54]	0.81 [0.64–1.02]	0.75 [0.63–0.89]	0.93 [0.71–1.24]
Use of antithrombotics	2.49 [1.59–4.12]	2.10 [1.58–2.83]	1.65 [1.35–2.01]	1.77 [1.30–2.46]
Use of insulin	1.12 [0.80–1.58]	1.17 [0.93–1.48]	1.21 [1.02–1.44]	1.26 [0.98–1.64]
Use of metformin	0.68 [0.50–0.92]	0.69 [0.56–0.85]	0.74 [0.63–0.87]	0.84 [0.66–1.09]
Use of sulphonylureas	1.18 [0.85–1.63]	1.23 [0.98–1.53]	0.93 [0.78–1.10]	0.85 [0.66–1.10]
Use of GLP-1 RA	1.60 [0.86–2.74]	1.40 [0.83–2.19]	1.07 [0.69–1.58]	0.93 [0.50–1.72]

hHF, hospitalization for heart failure; CV, cardiovascular; MACE, major adverse cardiovascular events; MI, myocardial infarction; SD, standard deviation; UACR, urinary albumin-to-creatinine ratio; GLP-1 RA, glucagon-like peptide-1 receptor agonists

After adjustments, the associations found at univariate analysis were confirmed, namely, both *gainers* and *losers* were at increased risk of hHF and the composite hHF or CV death, whereas they were neutral for MACE and non-fatal MI (Table 2). Of interest is also that male sex was a risk factor only for MACE and non-fatal MI, whereas age was a risk factor across all outcomes, and baseline weight was a risk factor only for hHF and hHF/CV death. Of the antihyperglycemic therapies, insulin was an independent risk predictor of non-fatal MI, while use of metformin was an independent negative risk factor for hHF, hHF/CV death, and MACE.

### CREDENCE

**Fig. 4:**
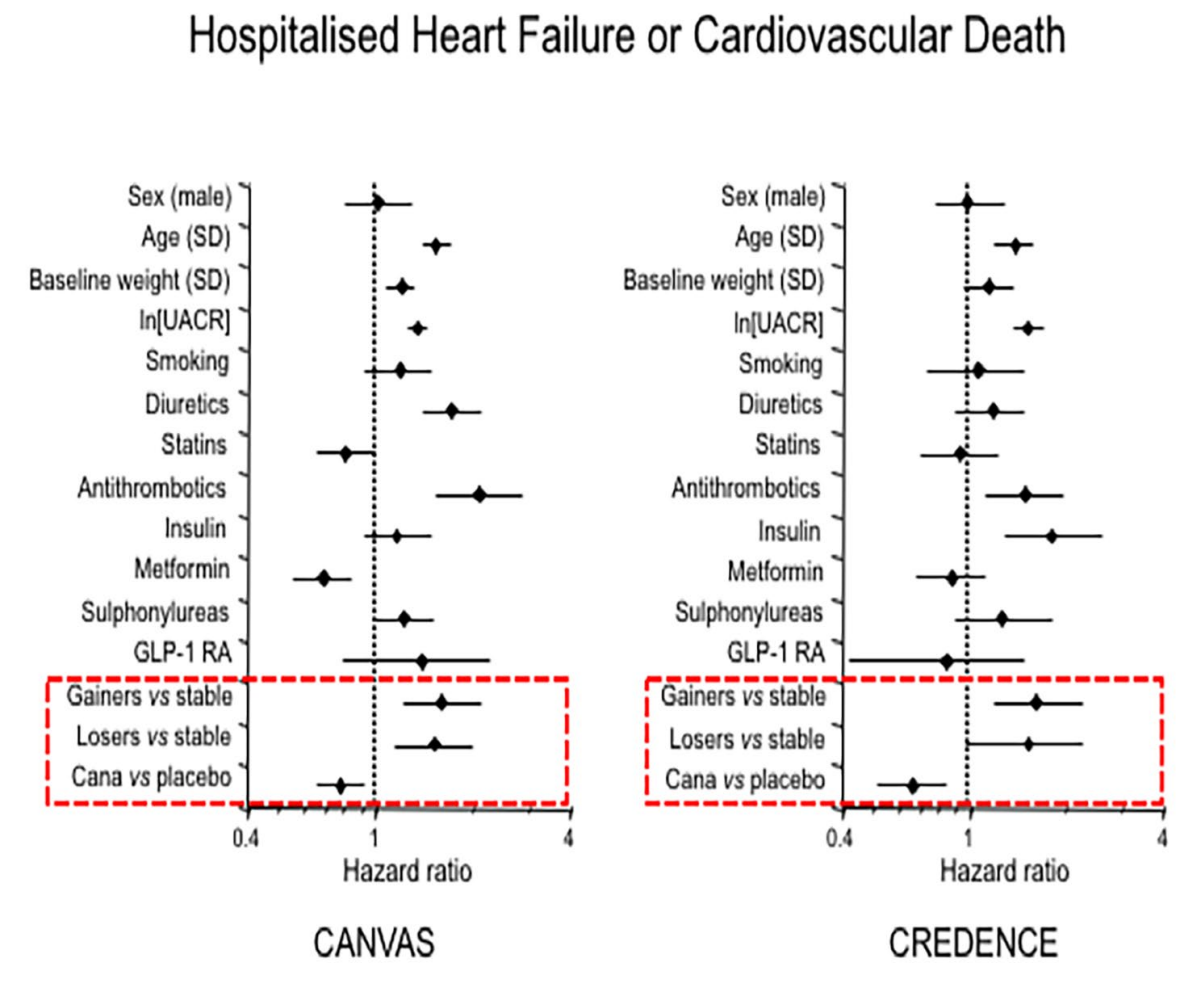
Multivariate Cox model for the endpoint of hospitalised heart failure or cardiovascular death in CANVAS and CREDENCE. Ln(UACR) = natural logarithm of urinary albumin-to-creatinine ratio

The replication cohort with complete data at week 78 consisted of 3,799 subjects (Supplemental Table 1); this cohort was altogether quite similar to that of CANVAS, except that by design patients had lower eGFR and much higher proteiniuria. In this cohort the time-course of BMI closely resembled that of the CANVAS participants (Fig. 1). Also like in CANVAS, baseline body weight was higher in both *gainers* and *losers* as compared to the weight *stable* group (Fig. 2). In CREDENCE, hospitalised congestive HF was the closest endpoint definition to the hHF of CANVAS. In univariate both *gainers* and *losers* had significantly worse hHF and hHF + CV death outcomes than weight *stable* subjects (Fig. 3). In bivariate Cox models including group and treatment, the interaction of these two terms was *p* = 0.11 for hHF and *p* = 0.64 for hHF + CV death. Furthermore, in multivariate Cox models (Table 3), the overall pattern of association of *gainers* with hHF and hHF/CV death resisted multiple adjustment, whereas this association fell short of statistical significance in *losers*; however, the HRs were of similar magnitude as in CANVAS (Tables 2 and 3; Fig. 4).

**Table 3:** Univariate and multivariate association of weight changes with major outcomes in CREDENCE. Entries are hazard ratio [95% confidence interval]

	hHF (n = 186)	hHF or CV Death (n = 264)	MACE (n = 299)	Non-fatal MI (n = 126)
*Univariate*				
**Gainers** ***vs*** **Stable**	1.71 [1.19–2.40]	1.82 [1.35–2.42]	1.29 [0.95–1.73]	0.88 [0.50–1.45]
**Losers** ***vs*** **Stable**	1.69 [1.00–2.69]	1.60 [1.02–2.41]	1.90 [1.29–2.70]	2.02 [1.14–3.45]
**Cana** ***vs*** **placebo**	0.57 [0.43–0.77]	0.63 [0.50–0.81]	0.81 [0.65–1.02]	0.94 [0.66–1.33]
*Multivariate*				
**Gainers** ***vs*** **Stable**	1.48 [1.03–2.10]	1.62 [1.19–2.16]	1.22 [0.89–1.64]	0.85 [0.48–1.41]
**Losers** ***vs*** **Stable**	1.50 [0.87–2.44]	1.53 [0.96–2.34]	2.03 [1.36–2.92]	2.15 [1.20–3.62]
**Cana** ***vs*** **placebo**	0.60 [0.44–0.81]	0.67 [0.52–0.86]	0.82 [0.65–1.03]	0.89 [0.63–1.28]
Sex (male)	0.91 [0.66–1.28]	0.99 [0.76–1.32]	1.29 [0.99–1.70]	1.13 [0.76–1.72]
Age (SD)	1.48 [1.26–1.75]	1.39 [1.21–1.60]	1.20 [1.06–1.36]	1.33 [1.09–1.62]
Baseline weight (SD)	1.29 [1.11–1.50]	1.15 [1.01–1.32]	0.95 [0.84–1.08]	0.94 [0.77–1.15]
Ln[UACR (mg/g)]	1.52 [1.29–1.80]	1.52 [1.33–1.76]	1.30 [1.15–1.48]	1.21 [1.00–1.47]
Smoking	1.23 [0.81–1.82]	1.07 [0.74–1.51]	0.90 [0.63–1.25]	1.27 [0.77–2.00]
Use of diuretics	1.34 [0.99–1.82]	1.19 [0.93–1.54]	1.08 [0.85–1.37]	1.28 [0.89–1.85]
Use of statins	1.33 [0.93–1.93]	0.94 [0.72–1.25]	0.97 [0.75–1.26]	1.62 [1.04–2.67]
Use of antithrombotics	1.50 [1.08–2.12]	1.49 [1.14–1.98]	1.65 [1.27–2.16]	1.58 [1.05–2.43]
Use of insulin	2.20 [1.47–3.35]	1.81 [1.30–2.55]	1.42 [1.05–1.94]	1.62 [1.02–2.63]
Use of metformin	0.96 [0.71–1.30]	0.88 [0.68–1.14]	0.87 [0.69–1.11]	0.89 [0.62–1.29]
Use of sulphonylureas	1.45 [1.01–2.08]	1.26 [0.92–1.71]	1.03 [0.76–1.38]	1.19 [0.75–1.84]
Use of GLP-1 RA	0.79 [0.36–1.53]	0.85 [0.43–1.50]	1.11 [0.61–1.84]	1.81 [0.84–3.43]

hHF, hospitalization for heart failure; CV, cardiovascular; MACE, major adverse cardiovascular events; MI, myocardial infarction; SD, standard deviation; UACR, urinary albumin-to-creatinine ratio; GLP-1 RA, glucagon-like peptide-1 receptor agonists

## Discussion

The main findings from our analysis are that (a) in patients with T2D and CVD, ‘extremes’ of weight gain or loss are independently associated with an excess of hospitalisations for HF or CV mortality, and (b) these ‘effects’ are detectable regardless of canagliflozin treatment. These results require specification.

Firstly, the larger changes in weight in both directions generally occurred in persons with a higher baseline body weight. This phenomenon, previously reported in nondiabetic cohorts (although with higher variations) [15], has been interpreted to reflect the fact that unstable weight characterises a common pool of individuals of heavier body size on a path to gain more (*gainers*) or trying to lose some (*losers*). Secondly, a higher baseline weight was a consistent, independent risk factor for hHF in both CANVAS and CREDENCE, which is compatible with the physiological notion that a larger preload is a further challenge to myocardial contractile performance in the failing heart [6]. Thirdly, the clinical phenotype of *gainers* as well as that of *losers* were surprisingly similar to that of *stable* subjects (except for more smoking in *losers* and higher albuminuria in both, Table 1); conspicuously, history of CVD, DKD or HF were of the same magnitude. As participants were not *a priori* stratified by category of weight change, the fraction of subjects on canagliflozin was highest in *losers* and lowest in *gainers*. Notably, however, the degree of weight change within each category was only slightly, though significantly, larger with canagliflozin as compared to placebo (Table 1), suggesting that the ‘placebo’ (or spontaneous) weight change predominated over the weight-reducing action of canagliflozin. While there is no information from the trials on whether the weight changes in the subjects classified as *gainers* and *losers* were diet- or drug-induced or truly spontaneous, it is important to stress that the corresponding categories were defined based on weight stability between 52 and 78 weeks. Thus, the observed weight changes were unlikely to be short-term swings or related to the diuretic effect of canagliflozin as they were achieved and maintained at least year-long. Fourthly, the risk associated with being either *gainers* or *losers* applied to hHF and hHF/CV death but not to MACE or non-fatal MI even after multivariable adjustment. Interestingly, this pattern of associations with outcomes was the same as that of canagliflozin treatment itself, i.e., significant protection against hHF and hHF/CV death but not against ischemic endpoints. Furthermore, in the multivariate Cox models including both comparisons between the weight changes groups and canagliflozin treatment, the hazard ratios for the latter were the same as those calculated in univariate analysis (Table 2). This does not support the possibility that these weight fluctuations were strongly ‘mediating’ the effect of the drug on outcomes.

Finally, baseline anti-hyperglycaemic therapy differed across weight category as *gainers* were using more insulin and less metformin, and use of sulphonylureas was less in both *gainers* and *losers* as compared to *stable* subjects. In most patients with type 2 diabetes, chronic insulin treatment and use of sulphonylureas induce weight gain [16, 17], while metformin treatment has been consistently associated with modest weight loss [18, 19]. Therefore, background antidiabetic therapy possibly contributed to the separation of *gainers* and *losers* from the *stable* category.

The interpretation of the multivariable Cox models (Fig. 4) is rather straightforward. In high-risk cohorts like CANVAS and CREDENCE, older age, higher baseline body weight and urine albumin excretion, and greater use of diuretics and antithrombotics were expected risk predictors for incident hHF/CV death while use of canagliflozin was protective (as previously documented [13, 14]). In these patient populations, over whom risk of HF and CV mortality looms large, a significant increase in body weight might raise the burden to the heart *via* the hemodynamic (greater intravascular volume and extracellular fluid and cardiac output [20]), neurohormonal (enhanced adrenergic tone [21]), and inflammatory [22] mechanisms that characterise excess body mass [23]. Importantly, this association was found despite weight gain not being that consistent, since in *gainers* it was 4.5 kg (corresponding to an increase of 5% in relation to their baseline body weight). In the Framingham Heart Study it was found that for every 1 kg/m^2^ increase in BMI, the risk of incident HF increased by 7% in women and 5% in men [24]. Another analysis of patients from the Framingham cohort study found that CV mortality increased by 7% for every two additional years lived with obesity [25]. In patients with type 2 diabetes, this risk is further enhanced by the presence of insulin resistance, leading to alterations in myocardial substrate metabolism and structure [26].

Less clear is the mechanism by which weight loss contributes to HF risk. Reverse causality may partly explain these findings, whereby patients who do not lose great amounts of weight might maintain their metabolic reserve and cope better with the catabolic state that characterises HF [27]. On the other hand, worsening HF itself is associated with weight loss and sarcopenia [5, 28]. Which mechanism – or combination of mechanisms – may have prevailed in the current cohorts is not possible to claim from the available data. This finding seems to be quite specific for HF, since previous reports showed how, in obese patients, even reductions in body weight lower than those reported here for *losers* (2.5–3.5 kg vs. 8–9 in the present study) achieve a significant decrease in risk of all-cause mortality [2]. In any case, the current results provide further grounds for the clinical recommendation that in patients with T2D and high *a priori* risk of heart failure a large unintended change in body weight in either direction should be carefully assessed regarding its proximal causes in view of individualised management.

Strengths of this work are the large sample size, the quality of the data (RCTs with centralised measurements and adjudicated outcomes), the analysis of multiple endpoints in a discovery and a replication dataset, the use of a purely statistical criterion (i.e., 10% tails of a distribution) to create weight change categories, and formal consideration of relevant confounders. That the associations between these significant weight fluctuations and outcomes was differential between hHF and MACE/nonfatal MI lends further credence to the finding. Limitations include the fact that the subgroup defined by weigh loss post-randomization is biased and confounded and results need to be interpreted with caution. Measures of adiposity were not available, therefore whether weight changes reflected a change in body composition could not be assessed [29] Another limitation is the (inevitable) fact that the pattern of associations here described may be different if different cutoffs for weight change are adopted, and that, due to limited number of events and multiple testing, some of the associations fall just short of canonical statistical significance. As a consequence, an operational cut-off for weight change to be considered potentially harmful could not be established. Further, despite the efforts to consider treatment effect in the models, the weight loss effect of SGLT2i may have hampered stratification of the different weight loss categories, as possibly indicated by the wide confidence interval for the risk of hHF/CV death, not reaching statistical significance in the CREDENCE population. Finally, the impact of background antidiabetic therapy requires further investigation. The finding of a somewhat different role of insulin vs. metformin in the multivariate analysis between CANVAS and CREDENCE (Fig. 4) may be spurious, particularly since antihyperglycaemic treatment suffers from a substantial prescription bias.

## Conclusions

Extremes of weight gain or loss were independently associated with an excess of hHF and cardiovascular death. This suggests that, in patients with T2D and high cardiovascular risk, large changes in body weight should be carefully assessed in view of individualised management.

## Electronic supplementary material

Below is the link to the electronic supplementary material.

Supplementary Material 1

## Data Availability

The data underlying this project can be obtained through the Yale University Open Data Access Project (http://yoda.yale.edu/) under data use agreement.

## List of abbreviations

BMI: body mass index
CI: confidence interval
CV: cardiovascular
CVD: cardiovascular disease
DKD: diabetic kidney disease
eGFR: estimated glomerular filtration rate
GLP-1 RA: glucagon-like peptide-1 receptor agonist
HbA_1c_: glycated haemoglobin A_1c_
HDL: high-density lipoprotein
hHF: heart failure hospitalisation
HR: hazard ratio
IQR: interquartile range
MACE: major adverse CV events
MI: myocardial infarction
RAAS: renin-angiotensin-aldosterone-system
RCT: randomised controlled trials
SD: standard deviation
SGLT2i: sodium-glucose cotransporter-2 inhibitor
T2D: type 2 diabetes
UACR: urine albumin-to-creatinine ratio
