# Supplementary material for: Extremes of both weight gain and weight loss are associated with increased incidence of heart failure and cardiovascular death: evidence from the CANVAS Program and CREDENCE
Source: Cardiovasc Diabetol. 2023 Apr 29;22:100. doi: 10.1186/s12933-023-01832-5 (PMC10149021; doi:10.1186/s12933-023-01832-5)
Supplement: Supplementary file 1 — Supplementary Material 1 [file 12933_2023_1832_MOESM1_ESM.docx]

**Table S1 – Clinical and Metabolic Characteristics of the CREDENCE Cohort by Weight Loss Category and Treatment.***

| **Grouping** | **Gainers (*G*)** | | **Stable (*S*)** | | **Losers (*L*)** | | ***p (G vs S)*** | ***p (L vs S)*** | ***p_Cana_*** |
| --- | --- | --- | --- | --- | --- | --- | --- | --- | --- |
| **N (% of group)** | **Placebo**  **363 (62)** | **Cana**  **220 (38)** | **Placebo**  **1417 (48)** | **Cana**  **1540 (52)** | **Placebo**  **97 (37)** | **Cana**  **162 (63)** | **-** | **-** | **-** |
| **BMI change (kg/m^2^)** | **+2.4** | **+2.1** | **-0.3** | **-0.8** | **-4.0** | **-3.7** | **<.0001** | **<.0001** | **<.0001** |
| **Weight change (kg)** | **4.7 [3.0]** | **5.0 [3.7]** | **-0.4 [3.1]** | **-1.5 [3.3]** | **-9.2 [4.8]** | **-8.6 [3.3]** | **<.0001** | **<.0001** | **<.0001** |
| **Percent weight change** | **+5.5** | **+6.1** | **-0.4** | **-1.6** | **-10.1** | **-9.7** | **<.0001** | **<.0001** | **<.0001** |
| *Clinical phenotype* |  | | | | | | | | |
| Sex (% M) | 65 | 63 | 67 | 66 | 68 | 69 | ns | ns | ns |
| Age (years) | 62 ± 9 | 60 ± 9 | 64 ± 9 | 63 ± 9 | 61 ± 9 | 62 ± 10 | <.0001 | .0076 | ns |
| Baseline BMI (kg/m^2^) | 31.5 ± 6.3 | 31.8 ± 6.8 | 30.9 ± 5.8 | 30.8 ± 5.5 | 35.5 ± 8.1 | 34.3 ± 7.3 | .0237 | <.0001 | ns |
| Baseline body weight (kg) | 87 ± 22 | 89 ± 22 | 86 ± 19 | 86 ± 19 | 101 ± 27 | 98 ± 24 | .0421 | <.0001 | ns |
| eGFR (mL^.^min^-1.^1.73m^-2^) | 56 ± 18 | 54 ±19 | 57 ± 19 | 57 ± 18 | 53 ± 19 | 57 ± 18 | .0080 | ns | ns |
| Type 2 diabetes duration (years) | 15 [10] | 14 [11] | 15 [12] | 15 [11] | 15 [14] | 14 [12] | ns | ns | .0465 |
| HbA_1c_ (%) | 8.37 ± 1.37 | 8.24 ± 1.31 | 8.21 ± 1.28 | 8.28 ± 1.29 | 8.20 ± 1.41 | 8.12 ± 1.32 | ns  n | ns | ns |
| Systolic blood pressure (mmHg) | 141 ± 18 | 139 ± 17 | 140 ± 15 | 140 ± 15 | 141 ± 18 | 140 ± 15 | ns | ns | ns |
| UACR (mg/g) | 1055 [1398] | 1078 [1854] | 864 [1290] | 863 [1286] | 1054 [2087] | 889 [1204] | 0.0002 | ns | ns |
| HDL cholesterol (mmol/L) | 1.10 [0.41] | 1.13 [0.45] | 1.10 [0.40] | 1.09 [1.39] | 1.03 [0.36] | 1.06 [0.48] | ns | ns | ns |
| Prior CVD (%) | 50 | 46 | 47 | 51 | 56 | 47 | ns | ns | ns |
| Prior MI (%) | 100 | 100 | 100 | 100 | 100 | 100 | - | - | - |
| Prior HF (%) | 100 | 100 | 100 | 100 | 100 | 100 | - | - | - |
| Smokers (%) | 13 | 14 | 13 | 15 | 11 | 22 | ns | ns | ns |
| Use of loop or non-loop diuretics (%) | 46 | 54 | 46 | 45 | 54 | 50 | .0038 | ns | .0054 |
| Use of antithrombotics (%) | 55 | 61 | 60 | 41 | 61 | 60 | ns | ns | ns |
| Use of statin (%) | 69 | 69 | 69 | 71 | 71 | 71 | ns | ns | ns |
| Use of RAAS inhibitors (%) | 100 | 100 | 100 | 100 | 100 | 100 | - | - | - |
| Use of ß-blockers (%) | 42 | 45 | 39 | 39 | 47 | 38 | .0514 | ns | ns |
| Use of insulin (%) | 74 | 75 | 62 | 64 | 62 | 64 | <.0001 | ns | <.0001 |
| Use of metformin (%) | 55 | 50 | 60 | 60 | 53 | 61 | .0027 | ns | <.0114 |
| Use of sulphonylureas (%) | 29 | 17 | 30 | 30 | 28 | 28 | .0057 | ns | <.0157 |
| Use of GLP-1 RA (%) | 6 | 5 | 3 | 4 | 7 | 3 | .0075 | ns | .0154s |

*entries are mean ± SD or median [IQR]; *p (G vs S)* = Gainers *vs* Stable; *p (L vs S)* = Losers *vs* Stable and *p_Cana_* = Cana *vs* Placebo by 2-way ANOVA or χ^2^ and Cochran-Mantel-Haenszel test. BMI, body mass index; eGFR, estimated glomerular filtration rate; UACR, urinary albumin-to-creatinine ratio; HDL, high-density lipoprotein; RAAS, renin-angiotensin-aldosterone-system; CVD, cardiovascular disease; HF, heart failure; MACE, Major Adverse Cardiovascular Events; MI, myocardial infarction.
